# Changes in Perceptions of First Responders After Witnessing a Drug Overdose: Individual and Contextual Variations Among People Who Use Opioids in West Virginia

**DOI:** 10.13023/jah.0503.03

**Published:** 2023-12-01

**Authors:** Kathleen L. Egan, Kelly Gurka, Alexandria Macmadu, Herb Linn

**Affiliations:** East Carolina University; University of Florida; Brown University; West Virginia University

**Keywords:** Appalachia, first responders, harm reduction, naloxone, opioid, overdose

## Abstract

**Introduction:**

Success of opioid overdose interventions involving first responders is dependent on the comfort level that bystanders have with first responders and their willingness to call for assistance. Positive or negative experiences with first responders following witnessing an overdose may influence a person’s willingness to call a first responder for assistance in the future.

**Purpose:**

The objective of this study was to examine changes in bystanders’ perceptions of first responders following witnessing an overdose attended by emergency medical services or a law enforcement official. It specifically explored perception changes among a sample of individuals residing in Appalachia who use prescription opioids nonmedically.

**Methods:**

Individuals from West Virginia who used prescription opioids nonmedically were interviewed to examine changes in perceptions of first responders following witnessing an overdose. The analytic sample (N = 50) consisted of participants who witnessed an overdose for which 911 was called and stayed until a first responder arrived. Chi-square contingency tables and ANOVA were conducted to assess relationships between individual and contextual characteristics with changes in perceptions.

**Results:**

Findings indicate that the majority (63%) had improved perceptions of first responders, 6% had diminished perceptions, and 24% were unchanged. Changes in perceptions varied by income, presence during substance use, and prior concerns about first responders.

**Implications:**

Individuals who reported experiencing a positive interaction with first a responder after witnessing an overdose may be more likely to call 911 during an overdose and support other interventions by first responders (e.g., referral to syringe service programs or treatment with medications for opioid use disorder).

## INTRODUCTION

Drug overdose, primarily driven by opioids, continues to be the leading cause of injury-related death in the U.S.^1^ The opioid crisis remains particularly acute in regions of rural Appalachia, such as West Virginia. In 2018, the opioid-involved death rate in West Virginia was 42.4 deaths per 100,000 persons—nearly three times the national average.^2^ Further, the rate of fatal overdose in West Virginia is predicted to have increased by over 45% from 2019 to 2020.^3^ This is exacerbated by limited access to harm-reduction services and treatment^4^–^7^ in the state and heightened stigma associated with substance use.^8^

First responders, such as emergency medical services (EMS) and law enforcement, are most often the first healthcare providers on the scene of a drug overdose. In the U.S., EMS personnel and law enforcement are often equipped with naloxone to reverse opioid overdoses at the location at which the overdose occurred.^9^–^11^ Success of interventions utilizing first responders is dependent on the willingness of individuals who have witnessed an overdose (or found the overdose victim) to call a first responder for assistance. Traditionally, fear of arrest and distrust in law enforcement and EMS have been the most cited reasons given by witnesses for either not calling or delaying calling for help.^12^–^15^ In 2017, a rapid ethnographic study conducted in two towns in West Virginia found that a small sample of community members who inject heroin perceived that first responders lacked compassion for individuals who used heroin and stated that worries about legal repercussions would deter them from seeking assistance with an overdose.^8^

Since 2007, 48 states in the U.S., including West Virginia, have adopted Good Samaritan laws.^16^–^18^ While Good Samaritan laws vary by state, they generally provide immunity from arrest, charge, or prosecution for a person who reports an overdose.^16^,^19^ Positive experiences with first responders, including an absence of adverse legal consequences, may influence perceptions of first responders and willingness to call a first responder in the future. This is increasingly important as overdoses with fentanyl require more than one administration of naloxone,^20^ and repeat overdoses are common.^21^,^22^

The objective of this study was to examine bystanders’ perceptions of first responders among a sample of individuals residing in West Virginia who use prescription opioids nonmedically and witnessed a drug overdose attended by a first responder. It further sought to examine the effect of witnessing an emergency medical service or a law enforcement official respond to a drug overdose on changes in perceptions of first responders as well as contextual factors associated with those changes in perceptions.

## METHODS

The current study is part of a larger feasibility study that examined the acceptability of overdose education and naloxone distribution programs in rural West Virginia. From August 2014 to March 2015 (n = 169), participants were recruited using respondent-driven sampling from three rural (as defined by the USDA’s Rural–Urban Commuting Area Codes^23^) Appalachian counties located in southern West Virginia. The three counties were selected based on elevated rates, higher than the statewide rate, of unintentional drug overdose fatality^24^ and the availability of community partners for collaboration. To be eligible for participation, individuals had to be at least 18 years of age, reside in one of the three target counties, and report engagement in nonmedical prescription opioid use at least five times in the previous 90 days. The interviews were multi-method, including both closed-ended and opened-ended questionnaire items, and averaged 45 minutes in duration. Data were collected via interviewer-administered paper questionnaires and were later entered by study personnel into a secure web-based database (Research Electronic Data Capture [REDCap]). Individuals who consented to participate were provided one $25 gift card for their time and one $10 gas card for transportation. The study was approved by the Institutional Review Board at West Virginia University.

### Recruitment

Respondent-driven sampling, an approach used to recruit hidden populations,^25^ was used to recruit individuals who have experience with nonmedical prescription opioid use. Community partners (i.e., people with lived experience, overdose prevention organizations, academics) who resided in the target counties were trained to recruit and interview participants. First, community partners recruited seeds, individuals with a network from which additional participants could be recruited, by identifying potential participants from substance use treatment programs and personal social networks. Following screening for eligibility, the trained community partners conducted structured, in-person interviews in private and semi-private natural settings, such as private offices and participants’ homes. After an interview was conducted, each seed, and subsequent participant, was given two referral coupons to distribute to eligible peers. For each referral coupon that was redeemed, the referring participant was compensated with one additional $15 gift card for up to two additional participants recruited. This process was completed until several recruitment chains, consisting of multiple waves of recruits, were produced.

### Sample Characteristics

**Figure 1:**
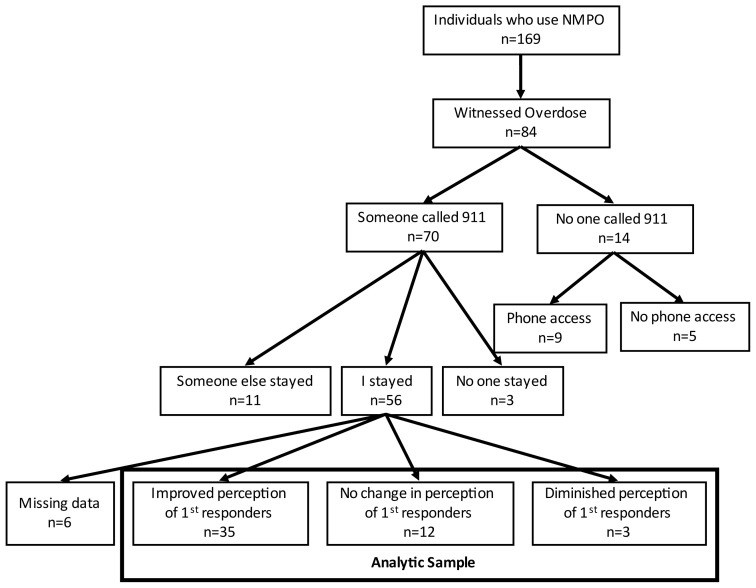
Consort Diagram NOTE: NMPO = nonmedical prescription opioid use

The analytic sample (Fig. 1; n = 50) consisted of participants who had witnessed a drug overdose (n = 84); called (or someone else had called) 911 (n = 70); stayed with the victim until a first responder arrived (n = 56); and responded to the item pertaining to perceptions of first responders (n = 50).

### Measures

Whether or not participants had witnessed a drug overdose was first assessed via the following question: “Have you ever witnessed a drug overdose?” Then, a determination of if 911 had been called was confirmed through affirmative responses to “What did you or others do to respond to the overdose? – Called 911”. Finally, participants who stayed with the victim were identified by affirmative responses, “yes, I did”, to the question “Did you or anyone stay with the person until he or she recovered or professional help arrived?”.

Participants’ perceptions of first responders were classified into one of two categories: (1) improved and (2) no improvement. If participants stated “yes, my views improved” in response to “Did your views of medical and/or public safety professionals change as a result of this experience?”, they were coded as ‘improved.’ If participants stated “yes, my views diminished” or “no” in response to “Did your views of medical and/or public safety professionals change as a result of this experience?”, they were coded as ‘no improvement.’ The intention was to have three mutually exclusive groups—improved, diminished, and no change—but small cell sizes required collapsing diminished perceptions and no change in perceptions into a single group.

Characteristics of the individual who witnessed the overdose and the context of the overdose were assessed. Individual characteristics included age (mean (standard error; SE)); gender (female v. male); race and ethnicity (coded as non-Hispanic white or another race/ethnicity); marital status (married, never married, or separated/divorced/widowed); education (coded as ≤ high school, high school diploma or GED, or > high school); employment (no v. yes); annual household income (coded as ≤ $15,000, $15,000–$25,000, or > $25,0000); ever overdosed (no v. yes); and number of overdoses witnessed (mean (SE)). Context of the last witnessed overdose consisted of location (coded as my house, victim’s house, someone else’s house, or other); number of people present (mean (SE)); relationship with overdose victim (coded as family or significant other, friend, acquaintance, or stranger); whether the participant was present when the person was using drugs (no v. yes); overdose included opioids (i.e., heroin or prescription; no vs. yes); whether the participant was worried about police or legal repercussions (no vs. yes); and whether the participant was concerned about what paramedics/doctors would say or do (no vs. yes). In the consort diagram (Fig. 1), for those who indicated no change in perceptions of first responders following the last overdose they experienced, we report prior concerns with first responders. For this purpose, we consolidated the two items pertaining to concerns with (1) “police or legal repercussions” and (2) “what paramedics/doctors would say or do” into a single item. If the participant indicated they had a concern about one or both, they were coded as having prior concerns. If they stated no for both, they were coded as having no prior concerns.

### Statistical Analyses

The analytic sample (N = 50) consisted of participants who had witnessed a drug overdose, called (or someone else had called) 911, and stayed with the victim until a first responder arrived. Descriptive statistics were first computed to describe the analytic sample, stratified by “improved” and “no improvement” in perceptions of first responders. Chi-square contingency tables were then used to assess associations between the individual/contextual characteristics and participant perceptions of first responders; two-sided Fisher’s exact tests were conducted given the small cell sizes. Continuous variables were analyzed using an ANOVA F test. All analyses were completed using SPSS 27 (Armonk NY: IBM Corp).

## RESULTS

Half (n = 84) of the individuals who reported nonmedical use of prescription opioids had witnessed a drug overdose. Of those who witnessed a drug overdose, at the last overdosed they witnessed, 67% (n = 56) called 911 and stayed with the victim until help arrived or the victim recovered. Among individuals who stayed with the victim, 89% (n = 50) provided their perceptions of first responders to the interviewer; six did not respond to the questions about first responders (Fig. 1).

About half of the participants included in the analysis were male (52%) and most were non-Hispanic white (74%), with a mean age of 38.5 years (SE = 1.6). There was an even distribution among those who were married (38.0%) and separated, divorced, or widowed (36.0%) at the time of data collection; about one-quarter had never been married (26.0%). The majority were unemployed (88.0%); did not graduate high school (34.0%) or only earned a high school diploma (56.0%); and had an annual household income of less than $15,000 (83.7%). Over half of the sample had never overdosed on a substance (69.4%), but the mean number of overdoses that they had witnessed in their lifetime was 2.7 (SE = 0.36).

Among those who stayed with the person who overdosed, 70% (n = 35) had improved perceptions of first responders, and 30% (n = 15) had no improvement. Only 6% (n = 3) had diminished perceptions of first responders, and 24% (n = 12) of laypersons’ perceptions of first responders did not change. Perceptions of first responders were not equally distributed across income categories (c^2^ (2, *N = 49*) 10.45, *p* < .01, Cramer’s V/phi = 0.46; Table 1). Individuals who had household incomes of less than $15,000 were significantly more likely to report improved perceptions of first responders compared to those with higher annual household incomes. Individuals who were present when the overdose victim was using substances were significantly more likely to report improved perceptions of first responders compared to those who were absent during substance use (c^2^ (1, *N = 50*) 6.82, *p <* .05, Cramer’s V/phi = 0.37; Table 2). Additionally, those who were concerned about what paramedics or doctors would say or do were significantly more likely to report improved perceptions of first responders (c^2^ (1, *N = 49*) 5.09, *p* < .05, Cramer’s V/phi = 0.32; Table 2). Perceptions of first responders did not vary by other individual or contextual variables.

## DISCUSSION

The key findings of this study include: (1) among people in the sample who witnessed overdose, 83% were willing to call 911 and 67% were willing to stay with the victim until help arrived or the victim’s condition improved; (2) perceptions of first responders improved for over half of participants who stayed with the victim and, among those whose perceptions did not improve, the majority had no prior concerns; (3) a higher percentage of participants in the lowest income bracket reported improved perceptions of first responders; (4) individuals who were present when the overdose victim was using substances were more likely than those absent during substance use to have improved perceptions; and (5) the proportion of individuals who had prior concerns about what paramedics or doctors would say or do was higher for those who reported improved perceptions compared to no improvement.

Among the participants who witnessed a drug overdose, at the last overdose they witnessed, 67% (n = 56) both called 911 and stayed with the victim until either help arrived or the victim recovered. These findings are consistent with the literature on bystander calls for medical assistance.^14^,^26^,^27^ While there is room for improvement, many participants were willing to call and stay with an overdose victim until a first responder arrived. It is important to note that, at the time of data collection, West Virginia did not have a Good Samaritan law, as their law came into effect in June 2015.^18^ The authors hypothesize that passage of the Good Samaritan law in West Virginia, along with proper use and awareness of the law, should increase the percentage of laypersons willing to call 911 and remain with the victim until help arrives.^19^

Seventy percent of the sample was found to have improved perceptions of first responders, and only 6% (n = 3) had diminished perceptions. While a small qualitative study conducted in two West Virginia towns identified concerns about first responders and legal repercussions,^8^ this study’s findings suggest that positive interactions with first responders may lead to improved perceptions of them. The discrepancies between these findings and the previous study^8^ may also be contributed to differences in study participants as well as study design. A mixed-method study design with qualitative data that contextualized quantitative would be beneficial to better understand how perceptions of first responders change.

The proportion of individuals who had a household income of less than $15,000 was found to be significantly different from individuals with a household income of $15,000–$25,000 in their perceptions of first responders, with a higher percentage in the lower income bracket reporting improved perceptions. Further, individuals who were present when the overdose victim was using substances were more likely than those absent during substance use to have improved perceptions of first responders. Individuals with lower socioeconomic status^28^ and who are present when substances are being used^12^–^15^ may anticipate that they are at elevated risk of consequences following an overdose response attended by a first responder. The authors hypothesize that positive interactions, or at minimum the lack of negative interactions, with first responders may have improved the perceptions of these laypeople who may be at higher risk of adverse legal action.

A final finding was that the proportion of individuals who had prior concerns about what paramedics or doctors would say or do was higher for those who reported improved perceptions compared to no improvement. Individuals whose perceptions of first responders improved may have had more opportunity for these perceptions to change in a positive direction due to prior beliefs. Due to small cell sizes, those who reported no change and diminished perceptions were combined into a group that did not have improved perceptions. The majority (80%) of this group consisted of individuals who did not change their beliefs. Among those who did not change their beliefs following the overdose event, 67% did not have prior concerns about first responders. These findings suggest that laypersons’ experiences with first responders may increase positive perceptions, which may influence future receptiveness to interventions delivered by first responders. Future research should further examine the exchange between laypersons and first responders to determine if specific actions or experiences are more likely to build trust and positive relationships.

There are several limitations that should be considered when interpreting the findings of this study. The details regarding the witnessed overdose event may not have been recalled accurately, especially if there was an extended period between the last witnessed overdose and the interview. Participants witnessed an average of 2–3 overdoses in their lifetime; thus, their perceptions of first responders may have been shaped by a previous overdose that was not assessed in this study. Perceptions of first responders may be attributable to events other than the overdose and vary by type of first responders. It is possible that views of medical professionals improved while views of public safety professionals (i.e., law enforcement) diminished or vice versa, but the present analysis is unable to distinguish between the two. The findings may not be generalizable to other populations given the use of non-random sampling to recruit a small regional sample in rural West Virginia. Further, those who reported no change and diminished perceptions had to be combined into a group that did not have improved perceptions due to a small sample size. People who had diminished perceptions may be different from those who had no change in their perspectives. The data used in this analysis were collected in 2014 and 2015, prior to the passage of the Good Samaritan law in West Virginia, and perceptions of first responders may have changed since this data was collected. However, the findings may be relevant to states that have not passed a Good Samaritan Law, have passed a Good Samaritan Law that has fewer legal protections than comprehensive laws, or have passed a Good Samaritan Law but have not increased community awareness.^29^

## IMPLICATIONS

Laypersons residing in West Virginia who report nonmedical use of prescription opioids were found to be willing to call 911 and stay with the victim until help arrived or the victim’s condition improved. Perceptions of first responders improved for over half of participants, and among those whose perceptions did not improve, the majority had no prior concerns about first responders. Given positive perceptions of first responders, laypersons residing in West Virginia may be more likely to call 911 during an overdose and support other interventions by first responders (e.g., referral to syringe service programs or treatment with medications for opioid use disorder). This is increasingly important as overdoses with fentanyl require more than one administration of naloxone,^20^ repeat overdoses are common,^21^,^22^ and new interventions utilizing first responders continue to be developed.^30^,^31^ In recent years, opioid overdose response teams, often consisting of a peer and paramedic and/or law enforcement officer, are being implemented in communities, including in West Virginia, to connect individuals who have survived an opioid overdose to harm reduction services or treatment.^30^–^33^ The inclusion of peer outreach workers, often individuals who have lived substance use experience, in response teams may result in improved interactions with first responders if they are able to build stronger rapport with overdose victims and bystanders. Research should continue to examine the impact of witnessing first responders attend to overdoses on attitudes and beliefs of bystanders given the sociopolitical changes.

**SUMMARY BOX**

**What is already known about this topic?**

Success of interventions utilizing first responders is dependent on the willingness of individuals who have witnessed an overdose or found the overdose victim to call a first responder for assistance. Traditionally, fear of arrest and distrust in law enforcement and EMS have been the most cited reasons given by witnesses for not calling or delaying calling for help.

**What is added by this report?**

These findings indicate that Appalachian residents who observed an opioid overdose attended by a first responder had improved perceptions of first responders and changes in perceptions varied by income, presence during substance use, and prior concerns about first responders.

**What are the implications for future research?**

These findings can be used to inform future research, policy, and practice pertaining to the delivery of substance use interventions delivered by first responders.

## Figures and Tables

**Table 1:** Individual characteristics of participants who witnessed an overdose, called 911, and stayed with victim, by perception of first responders following the last witnessed overdose (n = 50)

	Perceptions of First Responders		
	Improved (n = 35) N (%)	Diminished or No Change (n = 15) N (%)	*p*-value
**Demographics**			

Age (mean (SE))	37.0 (1.9)	41.8 (2.8)	0.17

Gender			
Female	17 (48.6)	7 (46.7)	1.00
Male	18 (51.4)	8 (53.3)	

Race/Ethnicity			
Non-Hispanic, white	32 (91.4)	13 (86.7)	0.63
Another race/ethnicity	3 (8.6)	2 (13.3)	

Marital status			
Married	14 (40.0)	5 (33.3)	0.23
Never married	11 (31.4)	2 (13.3)	
Separated/Divorced/Widowed	10 (28.6)	8 (53.3)	

Education			
≤ High school	9 (25.7)	8 (53.3)	0.09
High school diploma/GED	23 (65.7)	5 (33.3)	
> High school	3 (8.6)	2 (13.3)	

Employment			
No	29 (82.9)	15 (100)	0.16
Yes	6 (17.1)	0 (0.0)	

Annual household income			
≤ $15,000	33 (94.3)	8 (57.1)	<0.01
$15,000–$25,000	2 (5.7)	5 (35.7)	
> $25,0000	0 (0.0)	1 (7.1)	

**Personal Overdose Experience**			

Ever Overdosed			
No	25 (73.5)	9 (60.0)	0.50
Yes	9 (26.5)	6 (40.0)	

No. overdoses witnessed (mean (SE))	2.6 (0.5)	2.9 (0.5)	0.71

**Table 2:** Contextual characteristics of last witnessed an overdose among those who called 911 and stayed with victim, by perception of first responders following the last witnessed overdose (n = 50)

	Perceptions of First Responders		
	Improved (n=35) N (%)	No improvement (n=15) N (%)	*p*-value
Location			
My house	5 (14.3)	2 (13.3)	
Victim’s house	15 (42.9)	7 (46.7)	0.92
Someone else’s house	14 (40.0)	5 (33.3)	
Other*	1 (2.9)	1 (6.7)	

# of people present (mean (SE))	4.5 (0.3)	4.1 (0.4)	0.36

Relationship with victim			
Family or significant other	12 (35.3)	7 (50.0)	
Friend	20 (58.8)	5 (35.7)	0.22
Acquaintance	1 (2.9)	2 (14.3)	
Stranger	1 (2.9)	0 (0.0)	

Present when victim was using substances			
No	6 (17.1)	8 (53.3)	0.02
Yes	29 (82.9)	7 (46.7)	

Opioid-involved overdose			
No	5 (14.3)	1 (6.7)	0.65
Yes	30 (85.7)	14 (93.3)	

Worried about police or legal repercussions			
No	17 (48.6)	11 (73.3)	0.13
Yes	18 (51.4)	4 (26.7)	

Concerned about what paramedics or doctors would say or do			
No	18 (52.9)	13 (86.7)	0.03
Yes	16 (47.1)	2 (13.3)	

NOTES:

* Other responses included “parking lot,” “high school,” “car,” “drug house,” and “motel room”
